# Anti-inflammatory potential via the MAPK signaling pathway of *Lactobacillus* spp. isolated from canine feces

**DOI:** 10.1371/journal.pone.0299792

**Published:** 2024-03-27

**Authors:** Mi Ae Park, Mirieom Park, Hyun-Jun Jang, Sung Ho Lee, Yeong Min Hwang, Soyeon Park, Donghyun Shin, Yangseon Kim

**Affiliations:** 1 Department of Research and Development, Center for Industrialization of Agricultural and Livestock Microorganisms, Jeongeup-si, Republic of Korea; 2 Department of Animal Biotechnology, Jeonbuk National University, Jeonju-si, Republic of Korea; 3 Woogene B&G Co., Ltd., Gyeonggi-do, Hwaseong-si, Republic of Korea; 4 The Animal Molecular Genetics and Breeding Center, Jeonbuk National University, Jeonju-si, Republic of Korea; Universidad San Francisco de Quito, ECUADOR

## Abstract

Two probiotic candidates, *Lactobacillus reuteri* C1 (C1) and *Lactobacillus acidophilus* C5 (C5), which were previously isolated from canines, were evaluated in the present study. *L*. *reuteri and L*. *acidophilus* have anti-oxidant, anti-inflammatory, immune-enhancing, and anti-cancer properties and exhibit various probiotic effects in humans and animals. The strains C1 and C5 demonstrated good tolerance to acid and bile salt exposure, exhibited effective adhesion to HT-29 cell monolayer, and displayed sensitivity to antibiotics, thus affirming their probiotic characteristics. Moreover, C1 and C5 exhibited the ability to downregulate the expression of inducible NO synthase (iNOS), an immunomodulatory factor, leading to a reduction in NO production in lipopolysaccharide (LPS)-stimulated RAW 264.7 cells. These strains also demonstrated potent anti-inflammatory effects in LPS-stimulated RAW 264.7 cells, achieved through the augmentation of anti-inflammatory cytokine IL-10 expression and the inhibition of pro-inflammatory cytokine IL-1β expression. These anti-inflammatory effects of C1 and C5 were closely associated with the mitogen-activated protein kinase (MAPK) signaling pathway. The results of the present study suggest that the C1 and C5 probiotic candidates attenuate LPS-induced inflammation via the MAPK signaling pathway and the strains can be used as probiotics considering their anti-inflammatory potential.

## Introduction

Probiotics are “live microorganisms, which, when administered in adequate amounts, confer a health benefit on the host” [1]. Probiotics help balance and restore the gut microbiota, promote competition with pathogens, and maintain host immune homeostasis, intestinal barrier integrity, and immune function [2]. Probiotics are commonly utilized in clinical practice in several countries and can be obtained by consumers with or without a prescription [3]. Probiotics mainly comprise *Lactobacillus* or *Bifidobacterium* species, originating from microbiota in healthy host intestine, dairy products, or fermented foods. Probiotics also include other bacterial genera, such as *Enterococcus*, *Streptococcus*, and *Bacillus*, and the yeast *Saccharomyces*. *Lactobacillus* strains, commonly found in the intestine of healthy individuals, are among widely used probiotics [4] and exhibit anti-oxidant, anti-inflammatory, immune-enhancing, and anti-cancer properties [5–8]. Identification and isolation of novel probiotic strains, particularly *Lactobacillus* strains, with health-promoting benefits, have garnered interest in the industrial and medical fields [9].

*L*. *reuteri* exerts probiotic effects that modulate the host immune system [10], produces diverse antimicrobial compounds [11], and prevents diarrhea [12] and colitis [13]. *L*. *reuteri* strain C1, which we isolated from a canine in a previous study, was compared with human *L*. *reuteri* via whole-genome sequencing. Our results indicated that canine C1 strain is similar to human *L*. *reuteri* [14].

*L*. *acidophilus* is an animal and human symbiont and is widely used to manufacture dairy products and fermented foods, including health foods and yogurt, and various medicines [15]. *L*. *acidophilus* confers many probiotic benefits in humans and animals and aids in the prevention and treatment of diarrhea, reduction of cholesterol levels, modulation of the immune system, and suppression of cancer development [16, 17]. We previously isolated *L*. *acidophilus* C5 strain from a canine and compared it with other original isolates via whole-genome sequencing. We found that that numerous genes in canine C5 strain are involved in carbohydrate metabolism, probably because of animal domestication by humans [18].

Nitric oxide (NO) is an endogenous gas synthesized from L-arginine by NO synthase (NOS) and participates in numerous biological functions and signaling and serves as a metabolic regulator and effector molecule [19, 20]. There are three distinct isoforms of nitric oxide synthase (NOS): endothelial (eNOS), neuronal (nNOS), and inducible NOS (iNOS). eNOS and nNOS are continually expressed, whereas iNOS is activated in response to oxidative stress and under pro-inflammatory conditions [21]. NO enables intestinal digestion, secretion, and motility and maintains water transport and mucus production. NO at high levels induces mutagenesis, apoptosis, and DNA damage [22]. Nitrate oxide produced by dendritic cells and macrophages causes local and systemic inflammation. Chronic, low-grade systematic inflammation leads to impaired insulin action, insulin resistance, obesity, hypertension, and metabolic syndrome [23, 24]. Several probiotic *Lactobacillus* and *Weissella* strains reportedly inhibit the production of NO in RAW 264.7 cells [25, 26].

In recent years, studies have focused on the potential application of *Lactobacillus* probiotics for mitigating inflammatory responses [27]. *L*. *acidophilus* SMC-S095 exhibits inhibitory effects on the production of TGF-β1 and IL-23, as well as the phosphorylation of STAT3, resulting in reduced pro-inflammatory cytokine IL-17 levels influenced by T-helper 17 (Th17) cells [28]. Administration of *L*. *helveticus* NS8 has been found to increase the level of IL-10 in peripheral blood mononuclear cells (PBMCs) *in vivo* [29]. Moreover, it has been reported that *L*. *plantarum* 21 alleviated TNBS-induced colitis in rats by suppressing TNF-α, IL-1β, and NO levels in colon tissues while simultaneously increasing glutathione (GSH) and IL-10 levels [30].

Various ailments, such as inflammatory bowel disease, obesity, diabetes, cardiovascular disorders, cancer, and metabolic syndrome, are linked to inflammation [31]. The mitogen-activated protein kinase (MAPK) signaling pathway plays a pivotal role in regulating cell proliferation, cellular response to cytokines, inflammation, immune modulation, and apoptosis [32, 33]. Key components of the MAPK pathway encompass extracellular signal-regulated kinases 1/2 (ERK1/2), p38 MAP kinases, and c-JUN N-terminal kinase (JNK) [34, 35]. The MAPK signaling pathway is responsible for the immunomodulatory activities and anti-inflammatory properties of probiotic microorganisms [36, 37].

The primary aim of this study was to evaluate the characteristics of C1 and C5 probiotic strains. We aimed to investigate whether these strains, when exposed to lipopolysaccharide (LPS) and cultured in a RAW 264.7 cell line, could suppress the production of NO via the modulation of inducible nitric oxide synthase (iNOS) expression. Furthermore, we examined the potential anti-inflammatory properties of C1 and C5 by assessing their capacity to reduce the expression of pro-inflammatory cytokine IL-1β and enhance the expression of the anti-inflammatory cytokine IL-10. Additionally, we delved into the molecular mechanisms that underlie these anti-inflammatory effects, focusing on the MAPK signaling pathway in LPS-stimulated RAW 264.7 cells, derived from murine macrophages.

## Materials and methods

### Isolation and identification of bacterial strains

Two probiotics, C1 and C5, were isolated from canines in our previous studies [14, 18]. As a reference strain, we employed *Lactobacillus rhamnosus* GG, sourced from the Korean Collection for Type Cultures (KCTC, Korea).

### Acid and bile salt tolerance

To assess tolerance to acidic and bile salt conditions, we used the procedure outlined in a previous study with a minor modification to simulate the gastrointestinal system [38]. The pH of sterile phosphate-buffered saline (PBS) was adjusted to 2.5 and 7.0 using 1 M HCl for experimental and control treatments, respectively, to measure the tolerance of bacterial strains to acidic conditions. Approximately 1 × 10^7^ CFU/mL of the isolates was incubated for 2 h at 37°C after overnight culture of the bacterial strains [38, 39]. To assess the bile tolerance of the strains, isolate growth was monitored for 8 h at 37°C in deMan Rogosa Sharpe (MRS) broth (Difco, USA) containing 0.3% or 1% oxgall (Difco, USA). Subsequently, the culture was subjected to 10-fold serial dilutions and applied on agar plates, which were then incubated for 24 h at 37°C. The viable colony count was employed to determine acid and bile tolerance. Each experiment was carried out in triplicate.

### Cell adhesion assay

The ability of microbial cells to adhere to the intestinal lining was determined using HT-29 colonic carcinoma cells derived from the human small intestine, as adopted from a previous study with minor modification [39]. Monolayers of HT-29 cells were prepared in Dulbecco’s modified Eagle medium (DMEM) (Sigma, USA) supplemented with 10% fetal bovine solution (FBS) (Sigma, USA) in 24-well tissue plates (BD Biosciences, USA) at 1 × 10^5^ cells/well. The cells were incubated at 37°C for 2 h together with 2 × 10^7^ CFU/mL of cultured strains to test for adhesion. After incubation, the HT-29 cells were aspirated and washed three times with 1 × PBS to remove unbound microbial cells. The adherent cells were detached, and appropriate dilutions were prepared, followed by enumeration of viable colonies on agar plates in triplicate.

### Antibiotic sensitivity assay

We evaluated the antibiotic susceptibility of the isolated bacterial strains using the E-test minimum inhibitory concentration (MIC) technique, specifically employing E-test BioMérieux BIODISK (BioMérieux, France). We assessed these strains against 11 antibiotic strips with MIC ranges of 0.016–256 g/mL for amoxicillin, ampicillin, clindamycin, erythromycin, gentamicin, kanamycin, metronidazole, tetracycline, and vancomycin, and 0.016–32 g/mL for imipenem and trimethoprim-sulfamethoxazole. For the test, we inoculated the target strains, on MRS agar plates, before adding the E-test strips. Subsequently, the MRS plates were incubated at 37°C for an additional 24 h. Antibiotic sensitivity was assessed considering the MIC as the antibiotic concentration at the point where dense colony growth intersected with the strip. Each experiment was conducted in triplicate.

### NO assay

We employed separate six-well plates for seeding RAW 264.7 cells, ensuring a density of 5 × 10^5^ cells per well. These plates were then incubated for 24 h at 37°C in a 5% CO_2_ environment. Following this incubation period, the medium in each well was substituted with fresh DMEM devoid of antibiotics. Meanwhile, the bacterial strains were cultured for 24 h at 37°C, after which they were quantified and the concentration was adjusted to 5 × 10^7^ CFU/mL. Subsequently, the bacteria were suspended in 1 mL of antibiotic-free DMEM and promptly added to RAW 264.7 cell culture, which had been subjected to 1 μg/mL LPS treatment for 24 h (Enzo Life Sciences, USA) in six-well plates, maintained at 37°C with 5% CO_2_ [40, 41]. To determine the quantity of nitrite present, we treated the supernatant with an equal volume of Griess reagent (Promega, USA). Nitrite levels was measured using a microplate reader (Tecan, Switzerland).

### Quantitative RT-PCR analysis of inflammatory gene expression

For the experiment, six-well plates were seeded with RAW 264.7 cells, ensuring a seeding density of 5 × 10^5^ cells per well. These plates were subsequently incubated for 24 h at 37°C in an environment containing 5% CO_2_. Subsequently, the culture medium in each well was replaced with antibiotic-free DMEM. Simultaneously, the bacterial strains were cultured for 24 h at 37°C; after which, they were quantified and the concentration was adjusted to 5 × 10^7^ CFU/mL. The bacterial cells were then resuspended in 1 mL of antibiotic-free DMEM. These bacterial suspensions were added into six-well plates containing RAW 264.7 cells that had been treated with 1 μg/mL LPS for 4 h [42, 43]. To assess the expression of both pro- and anti-inflammatory cytokines, we employed quantitative reverse transcription polymerase chain reaction (RT-qPCR). For the RT-qPCR, we used AMPIGENE qPCR Green Mix Lo-ROX (Enzo, USA), per the manufacturer’s instructions. We examined IL-1β, IL-10, iNOS, and GAPDH mRNA levels. The RT-qPCR were performed using specific primers (Table 1). To standardize the mRNA levels in each sample, we used GAPDH as the internal control. The ratio of normalized mRNA to the samples was determined using the comparative Ct method.

**Table 1:** Sequence information of the primers used in the RT-qPCR assay.

Gene		Sequence (5’ → 3’)	Amplicon size	Accession number
IL-1β	F	CCTGGGCTGTCCTGATGAGAG	127bp	NM_008361
	R	CGGGAAAGACACAGGTA		
IL-10	F	TGGGTTGCCAAGCCTTATCG	118bp	NM_010548
	R	TTCAGCTTCTCACCCAGGGA		
iNOS	F	AGCAACTACTGCTGGTGGTG	72bp	NM_010927
	R	TCTTCAGAGTCTGCCCATTG		
GAPDH	F	GGCCTTCCGTGTTCCTAC	103bp	NM_001289726
	R	TGCCTGCTTCACCACCTTC		

### Western blot analysis

We examined the effect of C1 and C5 on the expression of MAPK pathway proteins, specifically ERK1/2 and JNK, to elucidate the molecular mechanism underlying the alleviation of LPS-induced inflammatory stress in macrophages. Before the treatment with C1 or C5 (5 × 10^7^ cells/mL) in the presence of LPS (1 μg/mL), Raw 264.7 cells were cultured at a density of 5 × 10^5^ cells per well in six-well plates. After a 30-min incubation period, the supernatant was removed, and the cell pellets were washed in PBS before being lysed. Subsequently, equal amounts of protein were separated using 4–12% Tris-Glycine protein gels (Thermo Fisher, USA). The separated proteins were then transferred onto nitrocellulose membranes for western blot analysis. To detect the specific proteins, the ERK1/2, phospho-ERK1/2, SAPK/JNK, phospho-SAPK/JNK, and GAPDH antibodies (Cell Signaling Technology, USA) were used at the concentrations recommended by the manufacturer. Loading of equal amounts of proteins samples was confirmed using parallel western blots of GAPDH. The intensity of each band was measured using Image Lab software (Bio-Rad, USA).

### Statistical analysis

Statistical analyses were carried out using the PRISM software (GraphPad Software, USA). Shapiro-Wilk test was used to test the normality of data. The one-way analysis of variance (ANOVA) was used to analyze statistical differences among multiple groups using Tukey’s honestly significant difference (HSD) test as the post-hoc test. *P*-values < 0.05 were considered statistically significant. All experiments were conducted in triplicate.

## Results

### Probiotic characterization

**Fig 1:**
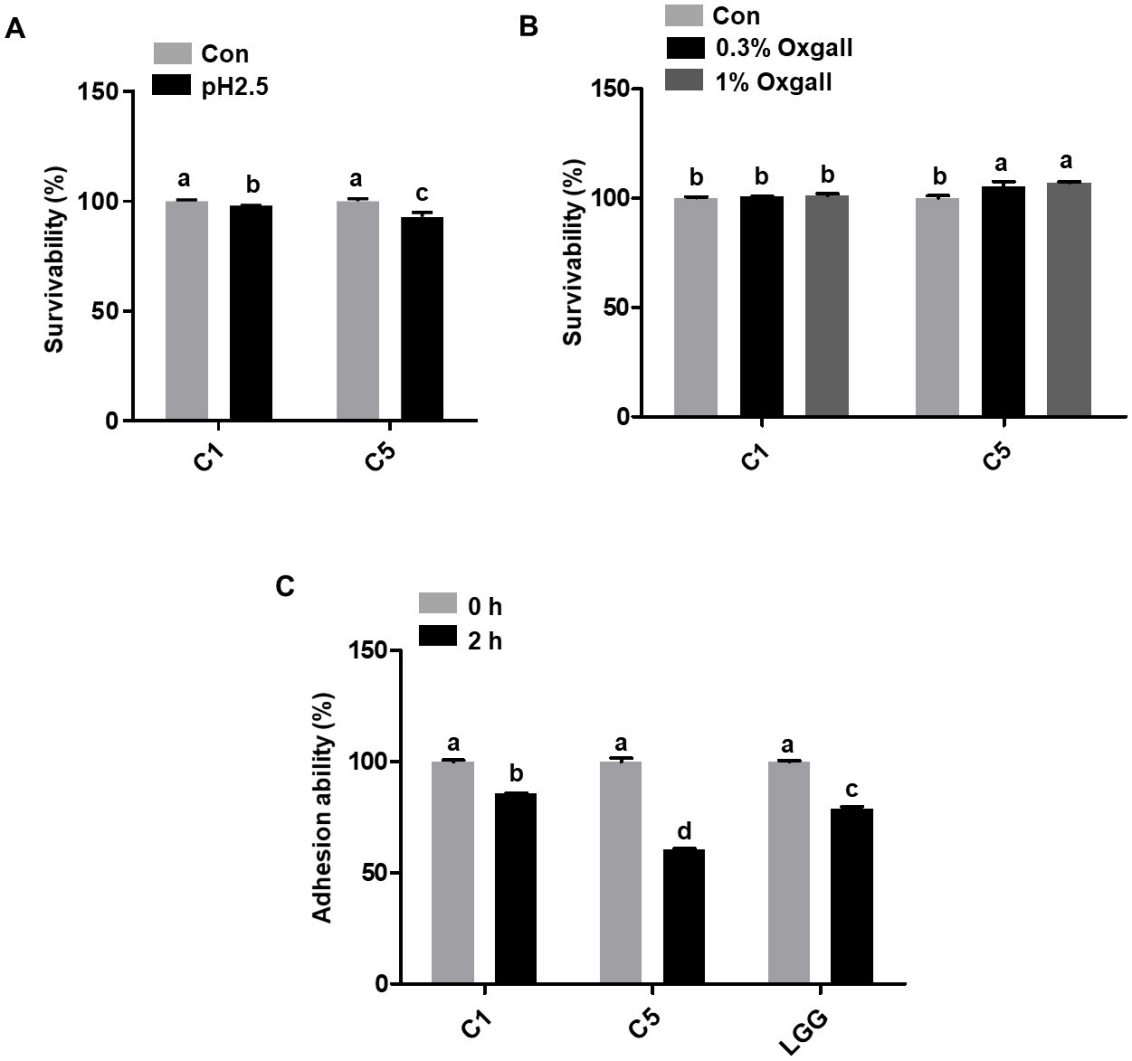
Acid and bile tolerance and intestinal adhesion activity. (A) Survivability test of bacterial strains at pH 2.5 and (B) 0.3% and 1% bile salt treatment for 2 h. (C) Adherence of the bacterial strains to human colonic carcinoma HT-29 cells. *Lactobacillus rhamnosus* GG (LGG) was used as a reference strain. Different letters indicate significant differences determined using Tukey’s honestly significant difference (HSD) test at *P* < 0.05. C1, *L*. *reuteri* C1; C5, *L*. *acidophilus* C5; Con, control.

The survivability of C1 and C5 was 98.1% and 92.9%, respectively, at pH 2.5 (Fig 1A). In the presence of 0.3% or 1% bile salts, the survivability of C1 was 100.6% and 101.1%, respectively, and that of C5 was 105.3% and 107%, respectively (Fig 1B). Adherence to human colonic carcinoma HT-29 cells was higher by C1 (85.78%) than by C5 (60.44%) and *L*. *rhamnosus* GG (LGG) control (78.74%; Fig 1C). The results indicate that the bacterial strains demonstrate tolerance to acidic and bile salt environments and possess the capability to adhere to intestinal cells.

Evaluation of sensitivity to commercial antibiotics revealed that C1 and C5 are resistant to kanamycin, metronidazole, and trimethoprim-sulfamethoxazole. In addition, C1 was resistant to vancomycin. The results suggest that the two strains evaluated in this study have different antibiotic-resistance spectra (Table 2, S1 Fig).

**Table 2:** Minimum inhibitory concentrations of antibiotics against the tested strains.

Antibiotic	Antibiotic sensitivity	
	C1	C5
Amoxicillin	≥0.38	≥0.5
Ampicillin	≥0.25	≥0.25
Clindamycin	≥0.032	≥4
Erythromycin	≥0.75	≥0.25
Gentamicin	≥4	≥4
Imipenem	≥0.047	≥0.064
Kanamycin	R	R
Metronidazole	R	R
Tetracycline	≥8	≥1
Trimethoprim-Sulfamethoxazole	R	R
Vancomycin	R	≥0.5

Antibiotic sensitivity is quantified as the minimum inhibitory concentration against the microbial strains and categorized as either resistant (R, ≥32 and 256 μg/mL) or susceptible to the antibiotic.

### NO production and iNOS expression in LPS-stimulated murine macrophages

**Fig 2:**
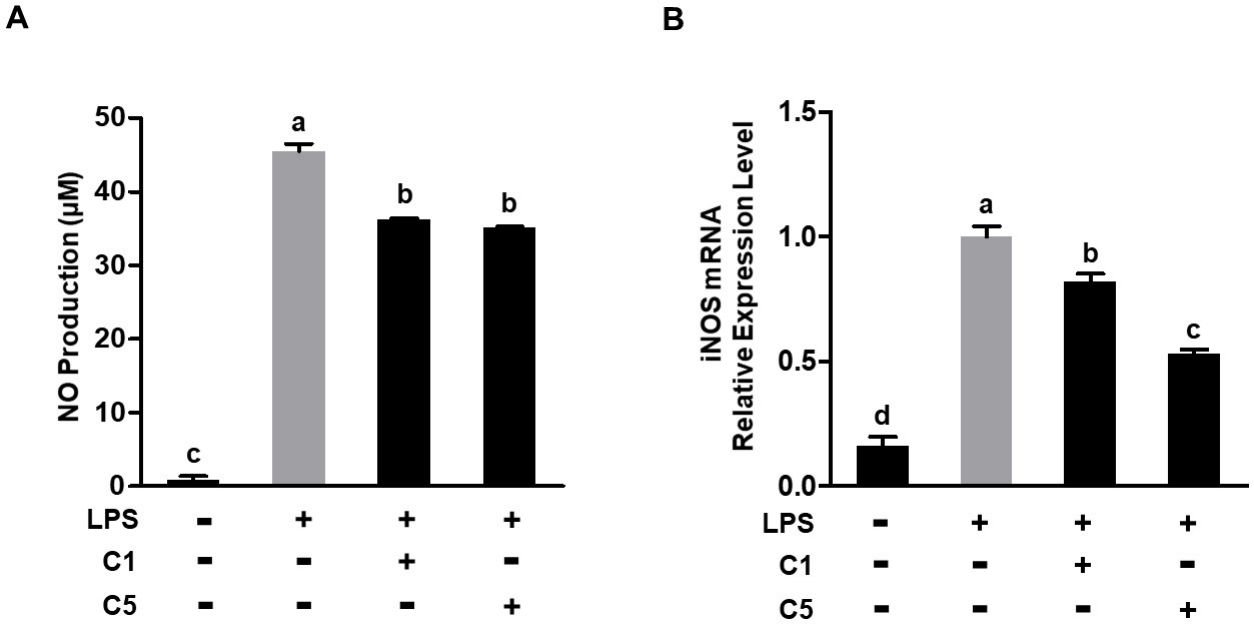
NO production and iNOS expression in LPS-stimulated RAW 264.7 cells. (A) NO production. (B) Quantitative real time PCR (RT-qPCR) analysis of mRNA expression of iNOS. Different letters indicate significant differences determined using Tukey’s honestly significant difference (HSD) test at *P* < 0.05. NO, nitric oxide; iNOS, inducible NO synthase; LPS, lipopolysaccharide.

We investigated the effect of C1 or C5 on NO production in LPS-stimulated RAW 264.7 cells, aiming to elucidate the anti-inflammatory potential of these strains. It was observed that LPS significantly upregulated NO production compared with that in the LPS-negative group (*P*< 0.001). However, treatment with either C1 or C5 resulted in a significant inhibition of NO production (*P*< 0.001, Fig 2A). To further explore the inhibitory effect of C1 and C5 on NO production, we examined whether the effect is associated with the changes in iNOS expression. As expected, LPS treatment led to a substantial increase in the mRNA expression of iNOS. However, treatment with C1 or C5 effectively suppressed the mRNA expression of iNOS in LPS-stimulated RAW 264.7 cells (Fig 2B). These results suggest that C1 or C5 treatment hampers NO production by suppressing the expression of iNOS.

### Anti-inflammatory responses in LPS-stimulated murine macrophages

**Fig 3:**
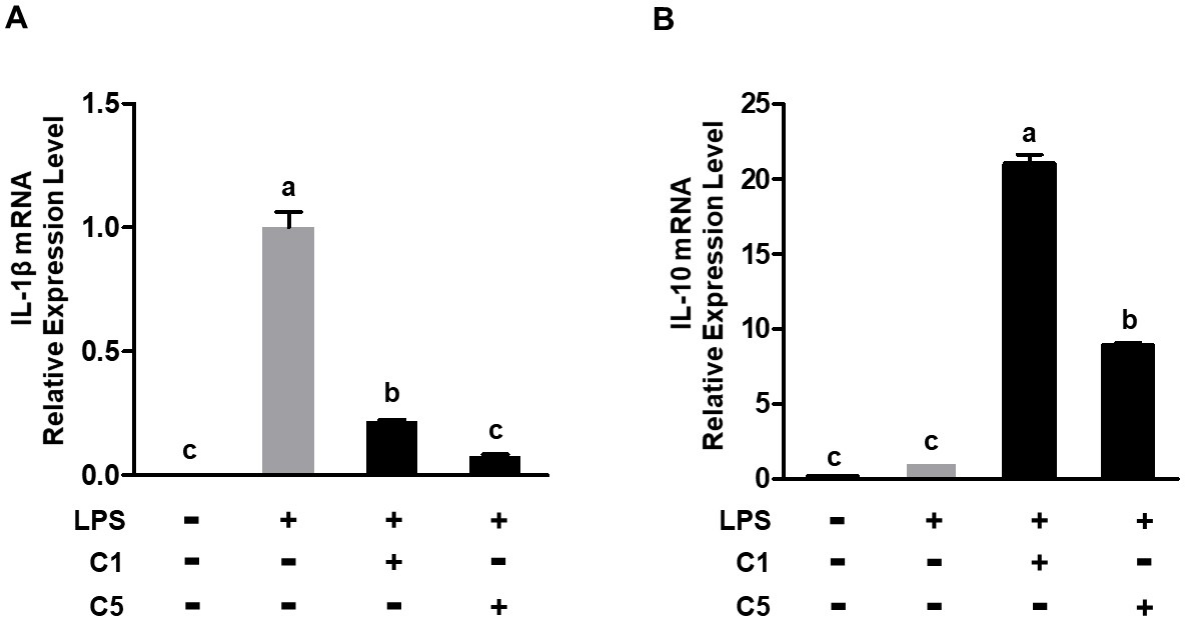
C1 and C5 treatment repressed pro-inflammatory cytokine expression and promoted anti-inflammatory cytokine expression. (A) mRNA levels of IL-1β and (B) IL-10 were assessed through RT-qPCR following to the treatment of RAW 264.7 cells with LPS (1 μg/mL) for 4 h and exposure to either the C1 or C5. LPS, lipopolysaccharide; IL-1β, interleukin-1β; IL-10, interleukin-10. Different letters indicate significant differences determined using Tukey’s honestly significant difference (HSD) test at *P* < 0.05.

Upon stimulation of RAW 264.7 murine macrophages cell line with LPS, a notable increase in IL-1β mRNA expression was observed compared with that in the untreated control. However, C1 and C5 treatment effectively suppressed the mRNA expression of the LPS-induced pro-inflammatory cytokine IL-1β (C1: 78% and C5: 92% vs. LPS; *P* < 0.001) (Fig 3A). Conversely, treatment with the C1 and C5 resulted in significant upregulation of the mRNA expression of the anti-inflammatory cytokine IL-10 (C1: 21-fold and C5: 8.9-fold vs. LPS; *P* < 0.001) (Fig 3B). These findings suggest that the C1 and C5 bacterial strains may exhibit anti-inflammatory properties when employed as probiotics.

### Regulation of MAPK activation

**Fig 4:**
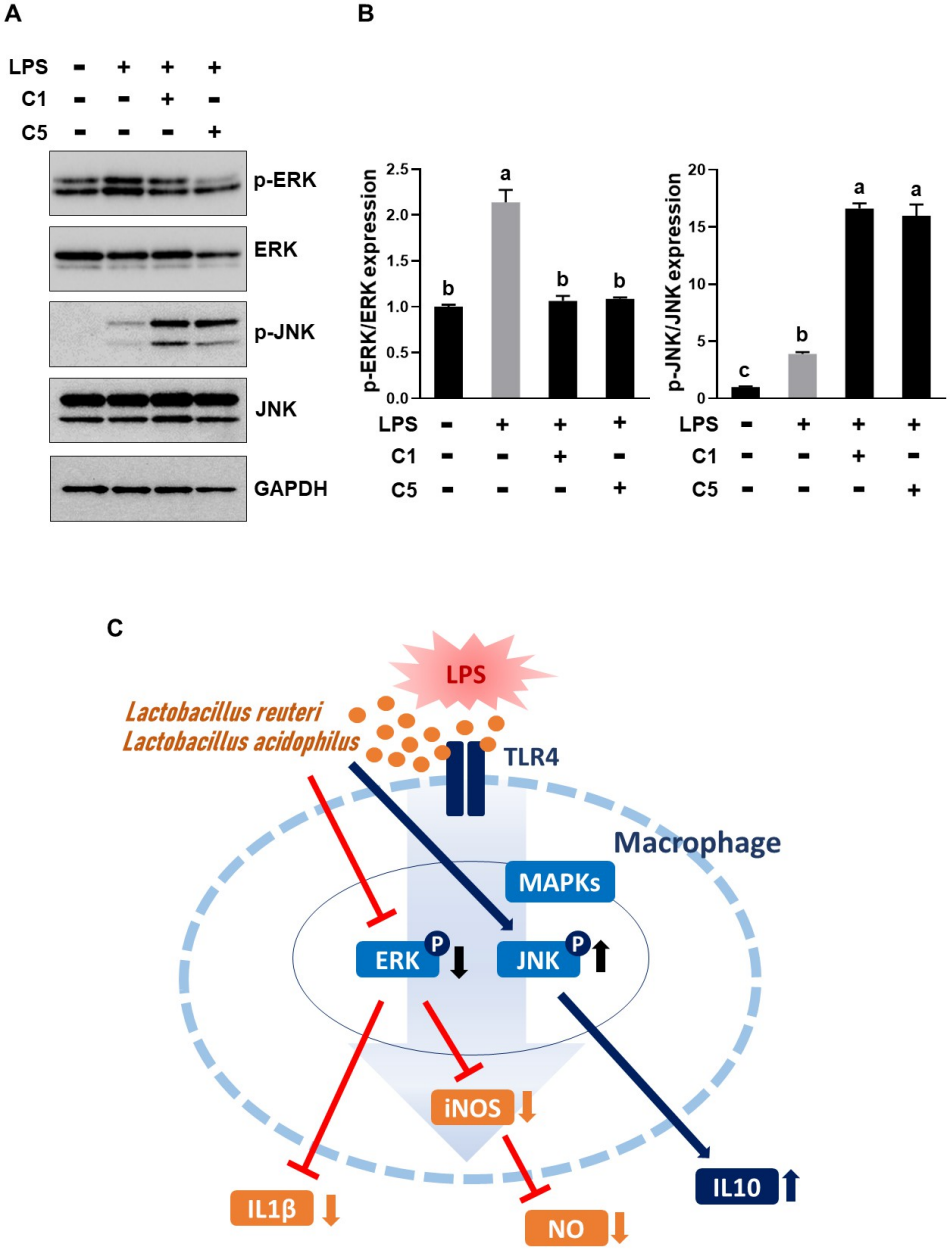
Regulation of MAPK activation. (A) Expression of MAPKs. ERK1/2, phosph-ERK1/2, JNK, and phospho-JNK protein levels were measured using western blotting in cells treated with or without 1 μg/mL LPS for 30 min followed by exposure to C1 or C5 strain and (B) relative quantification of band intensity. Different letters indicate significant differences determined using Tukey’s honestly significant difference (HSD) test at *P* < 0.05. (C) Schematic diagram showing the proposed mechanism underlying the anti-inflammatory effects of C1 and C5 in LPS-stimulated RAW 264.7 macrophages. ERK1/2, extracellular signal regulated kinases 1/2; JNK, c-JUN N-terminal kinase; MAPK, mitogen-activated protein kinase.

We investigated the molecular mechanisms underlying the observed anti-inflammatory effects of C1 and C5. The objective was to gain a deeper comprehension of how these strains regulate MAPK activation in RAW 264.7 cells following LPS stimulation. Upon LPS stimulation, we observed induction of the phosphorylation of MAPKs, including ERK1/2 and JNK. However, C1 and C5 treatment reduced ERK phosphorylation while concurrently inducing JNK phosphorylation in LPS-stimulated RAW 264.7 cells (Fig 4A and 4B). These findings strongly suggest that the anti-inflammatory properties of C1 and C5 are intricately linked to the modulation of MAPK activity (Fig 4C).

## Discussion

*Lactobacillus* species *L*. *reuteri* and *L*. *acidophilus* are considered probiotics [44]. *L*. *reuteri* C1 and *L*. *acidophilus* C5 strains have been characterized as commercial probiotic candidates based on their tolerance to acid and bile salts, as well as adhesion to intestinal cells.

*Lactobacillus* species have attracted considerable research interest owing their potential immunoregulatory functions [45, 46]. Two potential pathways of immunomodulatory action for *Lactobacillus* have been proposed: direct interactions with macrophages or macrophage activation via bacterial-released metabolites [45]. However, the molecular mechanisms via which *Lactobacillus* regulate immune responses are still unclear and require further investigation. This study revealed that the immunomodulatory activity of C1 and C5 is likely mediated via interaction with macrophages through co-culture of C1 and C5 with LPS-stimulated RAW 264.7 cells.

C1 and C5 were studied to elucidate their anti-inflammatory potential. C1 and C5 reduced NO production via downregulation of iNOS expression in LPS-stimulated RAW 264.7 cells. NO is a crucial biomarker in inflammatory responses and modulated by iNOS. LPS stimulates RAW 264.7 cells to induce iNOS expression by activating the MAPK and NF-kB signaling pathways, leading to the secretion of excessive NO. NO acts on vascular permeability and accelerates pain mediators, inflammatory infiltration, and monocyte release to the inflammatory site. To prevent or treat inflammatory diseases, inhibition of iNOS overexpression is crucial. The production of NO is closely associated with alterations in the expression levels of its synthesizing enzyme, iNOS [40, 47]. Researchers have shown that various probiotic *Bifidobacterium*, *Lactobacillus*, and *Weissella* strains inhibit NO production in RAW 264.7 cells or murine bone marrow-derived macrophages (BMDMs) [23, 40, 48].

Our study demonstrated that C1 and C5 treatment effectively mitigates the inflammatory reactions induced by LPS in RAW 264.7 murine macrophages. IL-1β plays a crucial role in immune responses at both the cellular and systemic levels. It serves as a prominent inflammatory cytokine that triggers the release of other inflammatory cytokines [49]. M2-type macrophages secrete high levels of IL-10, which plays roles in anti-inflammatory activities and damaged-tissue repair. In addition, *Lactobacillus* and *Bifidobacterium* can activate macrophages to induce anti-inflammatory cytokine IL-10 expression [50]. Our study revealed a reduction in the levels of the pro-inflammatory cytokine IL-1β and an induction in the levels of the anti-inflammatory cytokine IL-10 following C1 and C5 treatment. This finding is consistent with the results of previous studies, which have highlighted the beneficial effects of *Lactobacillus* species on host well-being [51–53]. Furthermore, previous research has established that *Lactobacillus* species possess the capacity to regulate the host immune system and mitigate inflammatory responses induced by LPS [41].

MAPKs are components of an important intracellular signaling system that regulates immune response transcription [54], although the specific MAPK signaling pathway related to probiotics is still unclear. Treatment with *Weissella cibaria* JW15 suppressed the activation of ERK1/2, JNK, and p38 MAPKs [40]. Research has revealed that selected lactic acid bacteria (LAB) probiotic isolates effectively decreased MAPK phosphorylation in LPS-stimulated RAW 264.7 cells [23]. On the contrary, *Lactobacillus* GG activates MAPKs and stimulates the expression of heat shock proteins in intestinal epithelial cells [51]. To elucidate the molecular mechanism underlying the anti-inflammatory properties of the chosen *Lactobacillus* probiotic isolates, we examined their effect on MAPK phosphorylation. C1 and C5 reduced ERK phosphorylation and induced JNK phosphorylation in LPS-stimulated MAPK phosphorylation. Treatment with the JNK inhibitor SP600125 reduced IL-10 mRNA and protein expression [55] and treatment with JNK inhibitor II reduced IL-10 protein expression, suggesting the involvement of miR-21-activated anti-inflammatory pathway via the JNK-IL-10 pathway [56]. Based on these findings, C1 and C5 may activate the IL-10 anti-inflammatory pathway via JNK phosphorylation. Further research is necessary to reveal the clear link between the MAPK pathway and anti-inflammatory properties of probiotics through the inhibition studies of MAPK pathway proteins and elucidate the precise molecular mechanisms that mediate the anti-inflammatory properties of probiotics via other signaling pathways.

## Conclusions

In the present study, we characterized two *Lactobacillus* probiotic strains isolated from canines and demonstrated their anti-inflammatory effects *in vitro*. *L*. *reuteri* C1 and *L*. *acidophilus* C5 can reduce NO production via inhibition of iNOS expression. In addition, C1 and C5 can stimulate macrophages to induce the expression of the anti-inflammatory cytokine IL-10 and reduce the expression of the pro-inflammatory cytokine IL-1β. The underlying molecular mechanisms include the MAPK signaling pathway, resulting in the regulation of MAPK phosphorylation. The present study highlights the molecular mechanisms via which the MAPK signaling pathway is regulated for the prevention of inflammation by probiotics.

## Supporting information

S1 Fig Antibiotic sensitivity assay images. (PDF)

S2 Fig Western blot raw images. (PDF)
