# Supplementary material for: Anti-inflammatory potential via the MAPK signaling pathway of Lactobacillus spp. isolated from canine feces
Source: PLoS One. 2024 Mar 27;19(3):e0299792. doi: 10.1371/journal.pone.0299792 (PMC10971663; doi:10.1371/journal.pone.0299792)
Supplement: S1 Fig — (PDF) [file pone.0299792.s001.pdf]

***L. reuteri* C1**

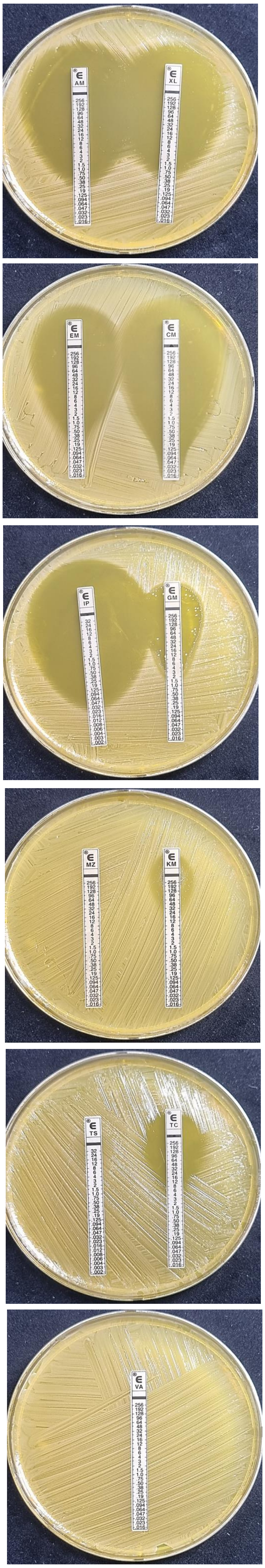

***L. acidophilus* C5**

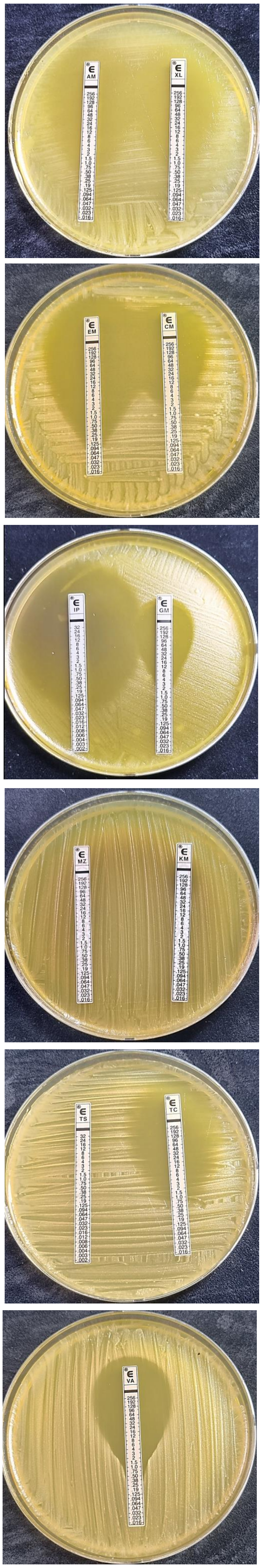

XL Amoxicillin

AM Ampicillin

CM Clindamycin

EM Erythromycin

GM Gentamicin

IP Imipenem

KM Kanamycin

MZ Metronidazole

TC Tetracycline

TS Trimethoprim-Sulfamethoxazole

VA Vancomycin
