# Supplementary figures and images for: Anti-inflammatory potential via the MAPK signaling pathway of Lactobacillus spp. isolated from canine feces
Source: PLoS One. 2024 Mar 27;19(3):e0299792. doi: 10.1371/journal.pone.0299792 (PMC10971663; doi:10.1371/journal.pone.0299792)

|     |   |   |   |   |
|-----|---|---|---|---|
| LPS | - | + | + | + |
| C1  | - | - | + | - |
| C5  | - | - | - | + |

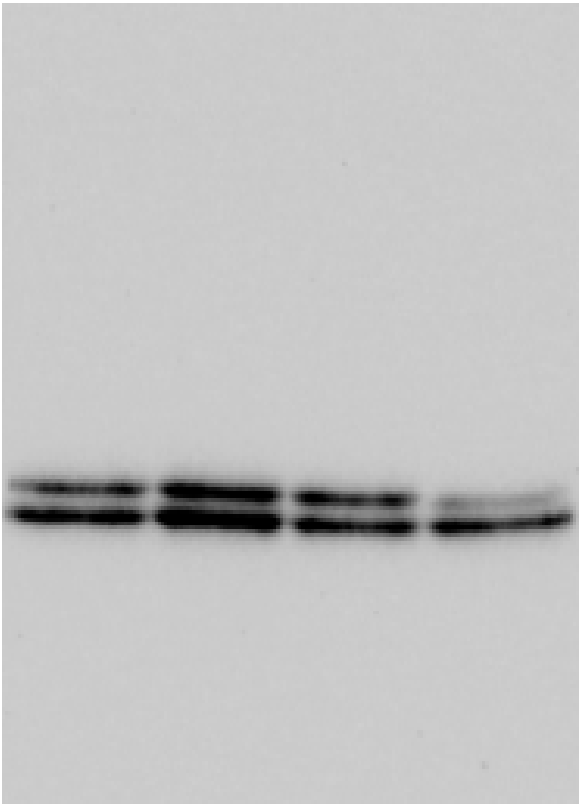

p-ERK

|     |   |   |   |   |
|-----|---|---|---|---|
| LPS | - | + | + | + |
| C1  | - | - | + | - |
| C5  | - | - | - | + |

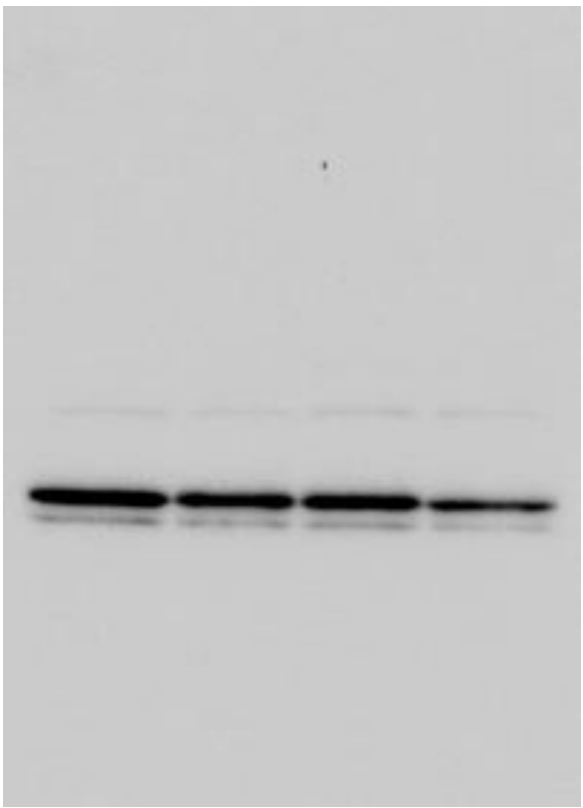

ERK

|     |   |   |   |   |
|-----|---|---|---|---|
| LPS | - | + | + | + |
| C1  | - | - | + | - |
| C5  | - | - | - | + |

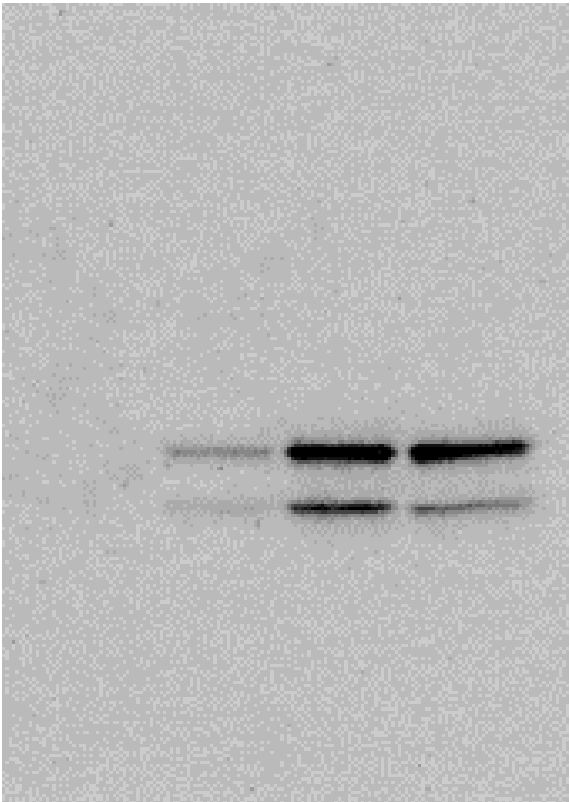

p-JNK

|     |   |   |   |   |
|-----|---|---|---|---|
| LPS | - | + | + | + |
| C1  | - | - | + | - |
| C5  | - | - | - | + |

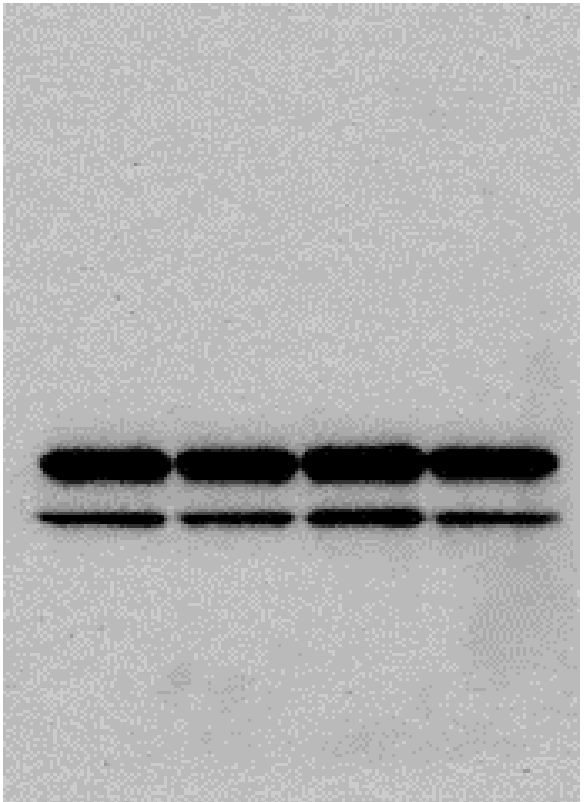

JNK

|     |   |   |   |   |
|-----|---|---|---|---|
| LPS | - | + | + | + |
| C1  | - | - | + | - |
| C5  | - | - | - | + |

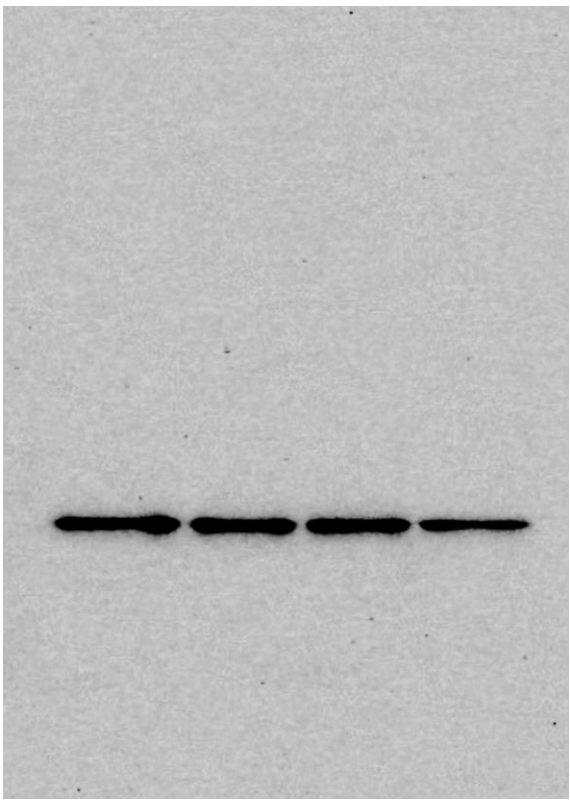

GAPDH

Supplement: S2 Fig — (PDF) [file pone.0299792.s002.pdf]
